# Incidence and characteristics of foot and ankle injuries among pilgrims during Hajj

**DOI:** 10.3389/fpubh.2026.1913192

**Published:** 2026-10-19

**Authors:** Ghadah Alsaleh, Bander Balkhi, Lamis Alabdullatif, Hala Aljishi, Fahad Alamri, Anas Khan, Fatemah Alsaleh

**Affiliations:** 1 Emergency Department, Neom Hospital, Tabuk, Saudi Arabia; 2 Global Center for Mass Gathering Medicine, MOH, Riyadh, Saudi Arabia; 3 Department of Clinical Pharmacy, College of Pharmacy, King Saud University, Riyadh, Saudi Arabia; 4 Department of Emergency Medicine, College of Medicine, King Saud University, Riyadh, Saudi Arabia; 5 Imam Abdulrahman Alfaisal Hospital MOH, Riyadh, Saudi Arabia

**Keywords:** foot injuries, Hajj pilgrimage, mass gatherings, prevalence, preventive measures

## Abstract

**Background:**

The Hajj pilgrimage represents one of the largest recurrent mass gatherings globally, posing unique injury-related public health challenges. Foot and ankle injuries are particularly common due to prolonged walking, crowd density, and environmental exposure.

**Methods:**

Methods: A retrospective observational study was conducted during Hajj 1444H, including approximately 1.8 million pilgrims. Injury data were extracted from healthcare facilities. Missing data were addressed using a distribution-based stochastic imputation approach designed to preserve the observed distribution of the available data. Incidence rates were calculated per 100,000 pilgrims.

**Results:**

A total of 943 foot injuries were identified (incidence: 52.4 per 100,000 pilgrims; 95% CI 49.1–55.8). Males accounted for 74.1% of cases and adults aged 31–64 years represented 50.2%. Superficial injuries were most common (57.3%), followed by ankle/foot injuries (14.8%) and fractures (8.2%). In an exploratory complete-case analysis, the overall distribution of injury categories differed by sex; however, this analysis included only 443 records and contained several sparse categories.

**Conclusion:**

Foot injuries during Hajj are common and largely preventable. Demographic findings should be interpreted cautiously because of substantial missingness and the unverifiable Missing At Random assumption. Targeted preventive strategies remain essential to reduce healthcare burden during mass gatherings.

## Introduction

The Hajj pilgrimage to Makkah is one of the largest annual mass gatherings worldwide, bringing together millions of individuals from diverse geographic, cultural, and demographic backgrounds. This convergence creates a uniquely complex public health environment characterized by extreme population density, logistical challenges, and heterogeneous health risks (1). Historically, Hajj has been associated with an increased burden of both communicable and non-communicable conditions, including infectious disease outbreaks, heat-related illness, and a wide spectrum of injuries (2, 3). In this context, injury epidemiology particularly musculoskeletal and soft tissue injuries has emerged as a critical but comparatively underexplored component of mass gathering medicine.

The rituals of Hajj involve sustained physical exertion over multiple days, including prolonged walking over considerable distances, often under conditions of crowd congestion and high ambient temperatures. These factors collectively predispose pilgrims to various health challenges. Among these, foot and ankle injuries are particularly prevalent, ranging from minor abrasions and blisters to more severe conditions such as fractures, sprains, and soft tissue damage (4, 5). The risk is further exacerbated by behavioral practices, including walking barefoot or wearing inappropriate or ill-fitting footwear, which remains common among pilgrims (6). These interacting environmental, behavioral, and physiological factors create a predictable yet preventable pattern of injury that warrants systematic investigation.

Foot injuries during Hajj have important clinical and public health implications. At the individual level, they can significantly impair mobility and limit the ability of pilgrims to complete essential religious rituals. At the system level, they contribute substantially to healthcare utilization, placing additional strain on already stretched medical services during peak periods (7). These injuries may also lead to secondary complications, particularly among vulnerable populations such as older adults and individuals with chronic conditions, including diabetes (8). Despite their apparent clinical relevance, foot injuries are often underreported or insufficiently characterized in large-scale surveillance systems, limiting the ability to design targeted interventions.

In recent years, increasing attention has been directed toward understanding the epidemiology of injuries during Hajj, given its implications for healthcare planning and resource allocation (9, 10). However, existing studies remain limited by methodological constraints, including incomplete data capture, variability in injury definitions, and insufficient adjustment for missing data. As a result, there remains a critical gap in reliably quantifying the burden, distribution, and determinants of foot and ankle injuries within this setting. Addressing this gap is essential for informing evidence-based preventive strategies and optimizing care delivery.

The risk of foot injuries is influenced by multiple interacting factors. Environmental conditions, such as extreme heat, hard walking surfaces, and overcrowding, increase the likelihood of both minor and traumatic injuries. Demographic factors, including age and sex, may also influence susceptibility and injury severity. In addition, behavioral factors—particularly footwear choice and physical preparedness—play a central role in determining injury risk (11). Understanding the relative contribution of these factors is essential for developing targeted, context-specific prevention strategies for mass gatherings.

Healthcare systems supporting the Hajj pilgrimage must operate under conditions of high demand, requiring rapid, efficient, and scalable responses to a broad range of medical needs. The burden of foot injuries necessitates coordinated service delivery, including primary care management, emergency services, and specialized orthopedic care for severe cases (7, 12). Accurate epidemiological data are therefore essential not only for clinical management but also for proactive planning, resource allocation, and policy development in mass gathering settings.

This study presents a comprehensive analysis of foot and ankle injuries among pilgrims during Hajj. By examining the incidence, distribution, and characteristics of these injuries, this study aims to enhance understanding of their epidemiology within a large-scale mass gathering context. The findings may help inform targeted preventive interventions, optimize healthcare delivery, and ultimately improve health outcomes and safety for pilgrims during future Hajj seasons.

## Materials and methods

### Study design and setting

A retrospective observational study was conducted to investigate foot and ankle injuries sustained by pilgrims during the Hajj season of 1444H. The study period spanned 30 days, corresponding to the official Hajj operational timeline from the 1st to the 30th day of the pilgrimage. Approximately 1.8 million pilgrims were included in the study population, representing a large-scale mass gathering with substantial healthcare demand.

The study was designed in accordance with the Strengthening the Reporting of Observational Studies in Epidemiology (STROBE) guidelines to enhance transparency and reproducibility.

The study adhered to the principles of the Declaration of Helsinki and received ethical approval from the Ministry of Health Ethical Committee (IRB #24-047E). Given the retrospective analysis of routinely collected de-identified data, the requirement for individual informed consent was waived by the Institutional Review Board.

### Data sources and collection

Data were obtained from the national Health Information System (HIS), which integrates clinical records from healthcare facilities operating during Hajj, including emergency departments and primary healthcare centers across key pilgrimage sites.

All pilgrims who presented to participating healthcare facilities during Hajj 1444H with a documented foot or ankle injury were eligible for inclusion. No sampling was performed; all eligible injury records available in the HIS during the study period were included to maximize case ascertainment.

For each eligible case, demographic characteristics (age, sex, and region/nationality, where available), injury-related information (injury type, anatomical location, and clinical classification), and contextual information (suspected mechanism or contributing factors, when documented) were extracted. Data extraction followed standardized HIS coding procedures to ensure consistency in the identification and classification of injury records.

### Outcome definitions

The primary outcome was a healthcare-attended foot or ankle injury recorded in the national Health Information System (HIS) during Hajj 1444H. A healthcare-attended injury was defined as an eligible foot or ankle injury documented for a pilgrim who presented to a participating emergency department or primary healthcare center during the study period. Injuries managed through self-care, informal care, or healthcare facilities not represented in the HIS were not captured.

Injury diagnoses were coded using the International Statistical Classification of Diseases and Related Health Problems, Tenth Revision, Australian Modification, Tenth Edition (ICD-10-AM). Based on the recorded diagnostic codes, injuries were assigned to the following mutually exclusive analytical categories: superficial skin injury; ankle and foot injury; wound; fracture; toe injury; muscular or tendon injury; dislocation; vascular or nerve injury; foreign-body injury; amputation; and unknown or unspecified injury. The ICD-10-AM codes assigned to each analytical category are presented in Supplementary Table S1.

These diagnostic categories were applied consistently throughout the descriptive analyses, and no additional severity-based reclassification was performed.

Data on foot injuries were comprehensively gathered from various healthcare facilities operating during Hajj, including emergency departments and primary healthcare centers. The data were extracted from the health information system (HIS), which contains detailed records of patient demographics, medical history, diagnoses, and treatment plans.

All pilgrims who presented to healthcare facilities during the Hajj season of 1444H with a foot injury and whose injuries were documented in the HIS were included in the study.

For each included pilgrim, the following information was extracted from the HIS:

- Demographics: Age, gender, and nationality of the injured pilgrims were documented where available.
- Injury characteristics: Detailed descriptions, including the type of injury (e.g., superficial skin injuries, fractures, wounds), specific location on the foot, and suspected causative factors, were recorded.

### Statistical analysis

Descriptive statistics were used to summarize demographic characteristics and injury patterns. Continuous variables (if applicable) were described using means and standard deviations, whereas categorical variables were summarized using frequencies and percentages.

Incidence measures were calculated as follows:

- Incidence proportion: number of injury cases per 100,000 pilgrims
- Incidence density: number of injury cases per 100,000 person-days, based on estimated cumulative exposure during the study period

Person-days were estimated using the standard epidemiological formula:

Person-days = Total pilgrims × Average length of stay.

where:

- Total pilgrims = approximately 1.8 million
- Average length of stay = 5.5 days Calculation:

1,800,000 pilgrims × 5.5 days = 9,900,000 person-days ≈ 10 million person-days

We used average length of stay (5.5 days) rather than the full 30-day operational period because not all pilgrims remain for the entire duration. This approach provides a more accurate estimate of exposure time for incidence density calculation (13, 14).

Confidence intervals for category-specific percentages were calculated using the Wilson method.

Descriptive demographic distributions were estimated using the prespecified missing-data procedure. In contrast, the analysis of injury type by sex was conducted as a complete-case analysis and included only records with observed values for both variables. Imputed sex values were not used in this subgroup comparison. Because complete cases represented 443 of the 943 eligible injury records, this analysis was considered exploratory.

The analysis of injury type by sex was restricted to records with observed information for both variables. Imputed sex values were not used in this inferential subgroup analysis. Because only 443 of the 943 eligible records were included, the analysis was considered exploratory and potentially susceptible to complete-case selection bias.

Injury categories were summarized descriptively by sex among records with observed values for both variables. Imputed sex values were not used in this analysis. No formal hypothesis test was performed because the complete-case subset represented less than half of the eligible records and several diagnostic categories contained small cell counts.

Given the exploratory nature of subgroup analyses and potential residual bias from missing data, emphasis was placed on effect size interpretation rather than solely on statistical significance.

Missingness was assessed separately for age, sex, and nationality or region. Missing demographic values were handled using distribution-based stochastic imputation, whereby missing values were assigned by random sampling from the observed distribution of the corresponding variable. This approach preserved the observed marginal distributions but did not incorporate relationships among variables or fully account for uncertainty arising from missing data. The resulting demographic estimates should therefore be interpreted cautiously.

All statistical analyses were conducted using SPSS software (IBM Corp., Armonk, NY, USA).

## Results

A total of 943 foot and ankle injuries were reported during the Hajj season. This corresponds to an incidence of 52.4 injuries per 100,000 pilgrims (95% confidence interval 49.1–55.8). Based on an estimated cumulative exposure of approximately 10 million participant-days, the incidence density was 9.4 injuries per 100,000 person-days.

### Demographic characteristics

Males accounted for 74.1% of cases, while females represented 25.9%. Adults aged 31–64 years constituted 50.2% of cases, followed by individuals aged 15–30 years (30.1%). Age information was missing for 10 patients (1.1%). Pilgrims from Saudi Arabia and Arabian Gulf countries comprised 51.2% of the cases, followed by those from other Arab countries (32.7%) (Table 1).

**Table 1:** Demographic characteristics of patients with foot injuries during Hajj 1444H.

Variable	Category	*n*	%
Gender	Male	699	74.1
	Female	244	25.9
Age group	0–14 years	104	11.0
	15–30 years	284	30.1
	31–64 years	473	50.2
	≥65 years	72	7.6
	Unknown/Missing	10	1.1
Region	Saudi & Arabian Gulf	483	51.2
	Arab countries	308	32.7
	Non-Arab Asia	109	11.6
	Other regions	43	4.5

### Injury classification and distribution

Table 2 presents the mutually exclusive diagnostic categories derived from the ICD-10-AM codes recorded in the Health Information System. Superficial skin injuries were the most frequently recorded category, accounting for 540 of 943 cases (57.3%). These were followed by ankle and foot injuries (140; 14.8%), wounds (87; 9.2%), fractures (77; 8.2%), and toe injuries (26; 2.8%). Less frequently recorded categories included muscular or tendon injuries, dislocations, vascular or nerve injuries, foreign-body injuries, and amputations. The diagnostic category was unknown or unspecified for 54 cases (5.7%). The overall distribution of diagnostic categories is shown in Figure 1. The overall distribution of injury types is presented in Figure 1.

**Table 2:** Distribution and cumulative incidence of healthcare-attended foot and ankle injuries by diagnostic category during Hajj 1444H.

Diagnostic category	*n*	% of recorded injuries	95% CI	Cumulative incidence per 100,000 pilgrims
Superficial skin	540	57.3	54.2–60.4	30.0
Ankle and foot injuries	140	14.8	12.6–17.1	7.8
Wound	87	9.2	7.4–11.2	4.8
Fracture	77	8.2	6.5–10.0	4.3
Toe injuries	26	2.8	1.8–4.0	1.4
Muscular and tendon injuries	6	0.6	0.2–1.3	0.3
Dislocations	5	0.5	0.2–1.2	0.3
Vascular and nerve injuries	4	0.4	0.1–1.0	0.2
Foreign body	2	0.2	0.0–0.7	0.1
Amputation	2	0.2	0.0–0.7	0.1
Unknown classification	54	5.7	—	—
Total	943	100	—	

**Figure 1:**
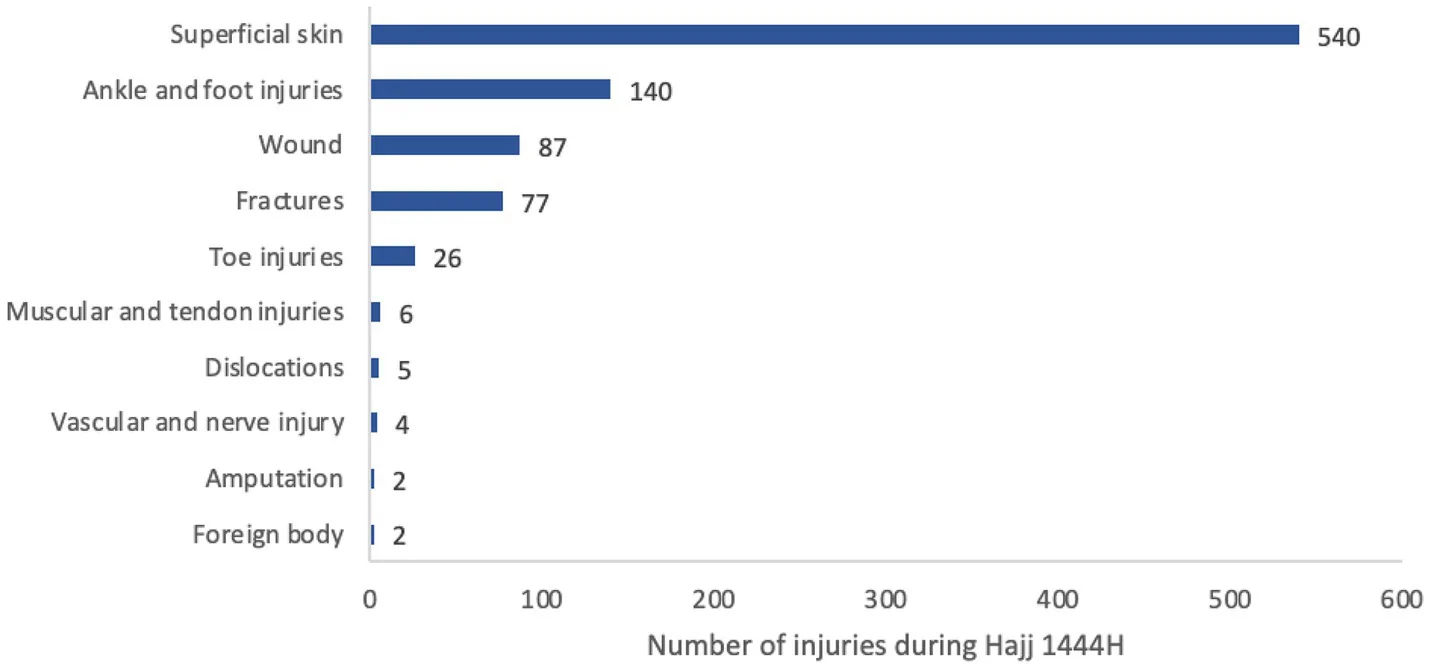
Frequency of classified foot injuries during the Hajj season 1444H.

### Descriptive distribution of injury categories by sex

The sex-stratified descriptive analysis was restricted to 443 records with observed information for both sex and injury category, representing 47.0% of the 943 eligible injury records. Imputed sex values were not used. Superficial skin injuries were the most frequently recorded category among both males and females. Within this complete-case subset, fractures represented 18.6% of injuries recorded among females and 10.0% among males, while wounds represented 8.0 and 12.4%, respectively. These percentages describe the distribution of recorded injury categories within the complete-case subset and should not be interpreted as sex-specific population risks. Because 500 eligible records were excluded and several diagnostic categories contained small numbers, the findings are exploratory and may be affected by complete-case selection bias (Table 3).

**Table 3:** Distribution of injury types by sex among complete cases with both variables observed (*n* = 443).

Injury type	Male (%)	Female (%)
Superficial skin	55.8	51.3
Wound	12.4	8.0
Fracture	10.0	18.6
Ankle and foot	15.8	15.9
Toe injuries	3.3	4.4
Vascular and nerve injury	0.6	0.0
Foreign body	0.6	0.0
Dislocations	0.9	0.0
Muscular and tendon injuries	0.6	0.0
Amputation	0.0	1.8

Values are presented as *n* (%), with percentages calculated within sex. Imputed sex values were not used. The analysis was exploratory and may be affected by complete-case selection bias.

## Discussion

This study provides a comprehensive epidemiological assessment of foot and ankle injuries during the Hajj pilgrimage using a large national dataset. Although the incidence proportion was relatively low in relation to the total pilgrim population, the absolute number of cases represents a substantial and continuous burden on healthcare services during a highly time-constrained mass gathering. This distinction between relative incidence and absolute burden is particularly important in the context of Hajj, where healthcare systems must respond rapidly to large volumes of acute presentations.

The predominance of superficial skin injuries observed in this study is consistent with previous reports from Hajj and other mass gatherings (7, 10). These injuries reflect the cumulative effect of prolonged walking, repetitive mechanical stress, and environmental exposure, including heat and hard walking surfaces. The reproducibility of this pattern across different studies suggests that superficial injuries constitute a predictable and system-level burden rather than sporadic events. Despite their relatively mild clinical severity, such injuries can substantially impair mobility and disrupt the completion of pilgrimage rituals, thereby amplifying their functional significance.

Gender differences in injury patterns were evident, with males representing the majority of cases, while a higher proportion of fractures was observed among females. The predominance of males is consistent with prior literature and may reflect higher exposure to mobility-related activities or differences in healthcare-seeking behavior (8, 12). The disproportionate occurrence of fractures among females represents a clinically relevant finding, which may be explained by a combination of physiological factors, including bone density variation, as well as differences in gait, balance, or footwear characteristics during pilgrimage activities (15, 16). However, given the reliance on complete-case analysis for gender-specific comparisons, these findings should be interpreted with appropriate caution.

The age distribution of injuries demonstrated that middle-aged adults constituted the largest affected group, a pattern that has been consistently observed in previous studies (10, 17). This likely reflects both the demographic composition of pilgrims and the physical demands of Hajj rituals, which require sustained exertion over several days. Importantly, while younger and older age groups represented smaller proportions, their inclusion highlights the need for risk stratification and age-specific preventive approaches tailored to varying levels of physical resilience and comorbidity profiles.

Environmental and behavioral determinants appear to play a central role in shaping injury patterns during Hajj. High ambient temperatures, prolonged walking distances, and dense crowd movement create conditions conducive to both repetitive stress injuries and acute trauma. Footwear practices emerge as a critical modifiable determinant, as inappropriate or poorly fitted footwear and barefoot walking increase exposure to friction, thermal injury, and mechanical stress. These findings are consistent with existing literature emphasizing the importance of biomechanical protection in high-exposure settings (11, 18–20).

From a health systems perspective, the observed distribution of injuries has important operational implications. The predominance of low-severity but high-frequency injuries suggests that a substantial proportion of healthcare encounters may be preventable through targeted public health interventions. These may include structured pre-Hajj educational campaigns, guidance on footwear selection, and strategies to reduce prolonged uninterrupted walking. At the same time, the presence of less frequent but clinically significant injuries such as fractures and amputations reinforces the need for maintaining advanced trauma care capacity within the Hajj healthcare system.

Special attention should be directed toward high-risk populations, particularly individuals with diabetes mellitus. Patients with diabetes are more susceptible to foot injuries and complications, including impaired wound healing and infection, which may significantly increase morbidity in the Hajj context (8). Preventive strategies for this group should include pre-travel medical optimization, patient education, and reinforcement of protective foot care practices.

Several limitations should be acknowledged. First, the study captured only injuries documented among pilgrims who presented to participating healthcare facilities. Injuries managed through self-care, informal care, or healthcare facilities not represented in the national Health Information System were not captured. Consequently, the reported cumulative incidence represents healthcare-attended foot and ankle injuries rather than all injuries occurring during Hajj and likely underestimates the overall injury burden. Under-ascertainment may have been greatest for mild superficial injuries, such as blisters and abrasions, because affected pilgrims may have been less likely to seek formal care. Therefore, although superficial skin injuries were the most frequently recorded diagnostic category, their proportion among all injuries occurring during Hajj may be higher than that observed in this healthcare-based dataset.

Second, the dataset lacked detailed clinical, environmental, and behavioral variables, including pre-existing conditions, diabetes status, footwear characteristics, walking exposure, injury mechanism, clinical severity, and treatment received. This limited our ability to identify independent risk factors, assess potential confounding, or determine whether particular injuries were directly attributable to specific environmental or behavioral exposures. Given the retrospective and descriptive design, the findings should not be interpreted as demonstrating causal relationships.

Third, demographic variables contained substantial missing data. The analysis assumed that these data were Missing At Random; however, this assumption could not be verified using the available retrospective records. Differential missingness according to injury severity, nationality, language barriers, healthcare facility, patient volume, or clinical urgency remains possible. For example, demographic information may have been less completely documented during urgent presentations, periods of high healthcare demand, or encounters involving communication barriers. If the probability of missingness was associated with these characteristics, the imputed demographic distributions may remain biased. Furthermore, the analysis of injury type by sex was restricted to records with complete information for both variables. Because this complete-case subset may not be representative of the full study population, the sex-specific findings should be regarded as exploratory and interpreted cautiously.

Fourth, individual-level information on pilgrims’ dates of entry into and exit from the surveillance population was unavailable, and daily counts of pilgrims within the healthcare surveillance catchment were not routinely collected. Consequently, the person-time denominator used to estimate incidence density was based on an assumed average duration of stay rather than observed individual-level follow-up time. Therefore, the reported incidence density should be interpreted as an approximate measure of injury occurrence and may be subject to measurement error. However, the cumulative incidence proportion based on the official number of pilgrims is likely to provide a more robust estimate of the burden of healthcare-attended foot and ankle injuries during Hajj.

## Conclusion

Foot and ankle injuries during Hajj represent a frequent and predictable component of healthcare utilization in mass gathering settings. While the incidence proportion is relatively low, the absolute burden is substantial due to the scale and intensity of the event.

The injury profile is dominated by superficial lesions associated with prolonged ambulation and environmental exposure, highlighting clear opportunities for prevention. Targeted interventions focusing on footwear optimization, structured health education, and protection of high-risk groups have the potential to meaningfully reduce injury burden and improve overall pilgrim safety.

## Data Availability

The data used for this study are available and will be shared by the corresponding author upon request.
