# Supplementary material for: Incidence and characteristics of foot and ankle injuries among pilgrims during Hajj
Source: Front Public Health. 2026 Oct 19;14:1913192. doi: 10.3389/fpubh.2026.1913192 (PMC13726619; doi:10.3389/fpubh.2026.1913192)
Supplement: Supplementary file 1 [file Table_1.DOC]

**Supplementary Table S1. ICD-10-AM code classification scheme used for healthcare-attended foot and ankle injuries during Hajj 1444H**

| **Analytical Category** | **Standardized ICD-10-AM Code Block** | **Inclusion / Technical Description** |
| --- | --- | --- |
| **Superficial skin injury** | S90.0–S90.9 | Abrasions, blisters, contusions, and superficial insect bites of ankle, foot, and toes (excluding open wounds and fractures). |
| **Open wound** | S91.0–S91.9 | Open wounds, lacerations, puncture wounds (with or without foreign body) involving ankle, foot, and toes. |
| **Fracture** | S92.0–S92.9 | Fractures of calcaneus, talus, tarsal, metatarsal, and phalanges (toes). |
| **Dislocation & Subluxation** | S93.0–S93.3 | Dislocations and subluxations of ankle, subtalar, midtarsal, metatarsophalangeal, and interphalangeal joints. |
| **Sprains, Strains & Ligamentous Injury** | S93.4–S93.6 | Sprains and strains of collateral, deltoid, calcaneofibular, and tarsal ligaments. |
| **Muscular & Tendon Injury** | S86.0–S86.9, S96.0–S96.9 | Strain, laceration, or traumatic rupture of muscles/tendons at lower leg/ankle level (including Achilles tendon S86.0) and foot level. |
| **Vascular & Nerve Injury** | S94.0–S94.9, S95.0–S95.9 | Traumatic injury to nerves (medial/lateral plantar, deep peroneal) and blood vessels (dorsalis pedis, posterior tibial) at ankle/foot level. |
| **Traumatic Amputation** | S98.0–S98.9 | Traumatic amputation of toe(s), midfoot, or complete foot at ankle level. |
| **Other & Multiple Specified Injuries** | S99.7–S99.8 | Specified multi-site or complex structural injuries not classified elsewhere. |
| **Unspecified Injury** | S99.9 | Unspecified injuries of foot and ankle, or incomplete diagnostic documentation. |
